# AI‐Enhanced Vibrational Capsule for Minimally Invasive Detection of Abnormal Bowel Tissue

**DOI:** 10.1002/advs.202517936

**Published:** 2026-02-17

**Authors:** Xizheng Fang, Yao Yan, Kenneth Omokhagbo Afebu, Andrew Bickerdike, Bo Tian, Yang Liu, Shyam Prasad

**Affiliations:** ^1^ Exeter Small‐Scale Robotics Laboratory Engineering Department University of Exeter Exeter UK; ^2^ School of Aeronautics and Astronautics University of Electronic Science and Technology of China Chengdu China; ^3^ Royal Devon University Healthcare NHS Foundation Trust Exeter UK

**Keywords:** artificial intelligence, biomechanical lesion detection, bowel cancer, minimally invasive diagnosis, one‐class support vector machine, vibration‐assisted capsule endoscopy

## Abstract

Colorectal cancer remains one of the leading causes of cancer mortality, and its prognosis is strongly dependent on early detection. Conventional diagnostic approaches, such as colonoscopy and capsule endoscopy, primarily rely on visual inspection and often fail to capture subsurface or biomechanical abnormalities. To address this limitation, an AI‐enhanced vibration‐assisted capsule is developed for in situ biomechanical sensing of bowel tissues. The self‐contained capsule integrates an eccentric vibration motor, triaxial accelerometer, power supply, and wireless communication unit within a swallowable format. During operation, motor‐induced oscillations deform surrounding tissue, and the resulting acceleration responses are wirelessly transmitted for real‐time analysis. Experiments conducted on ex vivo porcine colon specimens and a soft pneumatic in vitro colon simulator demonstrate that vibration‐induced signals capture reproducible mechanical contrasts between normal tissue and lesions of varying relative stiffness. A one‐class support vector machine, trained exclusively on normal tissue data, is employed to classify abnormal tissues without requiring extensive annotated datasets. Complementary dynamic modeling further identifies equivalent stiffness parameters, correlating lesion stiffness with abnormal classifications. This work demonstrates a minimally invasive, AI‐driven modality for non‐visual colorectal cancer detection, establishing the potential of vibration‐assisted capsules to provide real‐time, patient‐friendly screening and to complement existing imaging‐based diagnostics.

## Graphical abstract

A fully integrated vibration‐assisted capsule is presented for the minimally invasive detection of bowel lesions. The capsule incorporates a wireless sensor and an eccentric motor to probe tissue mechanics in situ. By coupling triaxial vibration signals with AI‐based classification and analytical modeling, the system enables early, non‐visual identification of abnormal tissue, offering a pathway toward intelligent diagnostic capsules.

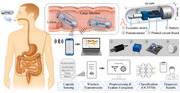

## 1 Introduction

Gastrointestinal health is increasingly recognized as a critical determinant of systemic homeostasis and long‐term disease prevention. Conditions such as colorectal cancer [1], inflammatory bowel disease [2], and irritable bowel syndrome [3] contribute substantially to the global disease burden. Among these, colorectal cancer remains a leading cause of cancer‐related mortality, with prognosis strongly dependent on stage at diagnosis [4, 5]. Early and minimally invasive detection is therefore essential for improving survival and reducing healthcare costs [6, 7]. This imperative is especially urgent for early neoplastic or precancerous lesions, which often present with vague or subclinical symptoms and are difficult to detect using conventional methods [8, 9].

Current diagnostic techniques such as colonoscopy, sigmoidoscopy, and capsule endoscopy rely primarily on visual inspection of the luminal mucosa [10, 11, 12]. While these approaches provide high‐resolution imagery, they offer limited sensitivity to subsurface or biomechanical alterations that accompany pathological remodeling [13, 14]. Recent advances in AI‐augmented gastrointestinal diagnostics have also leveraged spectrally enriched endoscopic representations, such as spectrum‐aided vision, hyperspectral or narrow‐band emulation, to improve lesion visibility and classification performance [15, 16, 17], reflecting a broader trend toward enhanced vision‐based representations that can complement biomechanical sensing. Subtle lesions, including fibrotic and early neoplastic changes, may still be missed under appearance‐based inspection [18]. Moreover, these methods demand extensive bowel preparation and specialized personnel, limiting their scalability for population‐wide screening [19, 20]. Tissue biopsy remains the gold standard, but is invasive, labor‐intensive, and incompatible with real‐time, distributed sensing across the gastrointestinal tract [21, 22]. Therefore, these limitations highlight the need for next‐generation, non‐visual, biomarker‐sensitive technologies capable of probing the mechanical and structural hallmarks of disease [23, 24].

Mechanical biomarkers such as stiffness, elasticity, and viscoelastic damping are increasingly recognized as valuable indicators of early pathological change. These properties capture processes such as tumor invasion, fibrosis, and inflammation, which often precede visible surface alterations. For example, Bickerdike et al. [25] combined vibrating microrobots with laser speckle contrast imaging to generate high‐resolution elasticity maps of bowel tissue, enabling real‐time tumor margin delineation without biopsy. Complementing this direction, Kim et al. [26] reported a mucosa‐interfacing capsule for in situ elasticity sensing that uses a magnetically actuated cantilever palpation unit with onboard magnetic and strain sensors and Bluetooth Low Energy telemetry; the device was validated on phantoms and ex vivo porcine colon, underscoring the feasibility of minimally invasive mechanical assessment in the gastrointestinal tract. In parallel, Traverso and colleagues reported a first‐in‐human trial of a vitals‐monitoring capsule that continuously tracked respiratory and cardiac signals from within the gastrointestinal tract [27]. Nan et al. [28] introduced a battery‐free, tissue‐adhering robotic interface for chronic gut electrostimulation, while Qiu et al. [29] reviewed soft integrated strain, pressure, and impedance sensors for bio‐interfacing in soft robotics. More recently, Srinivasan et al. [30] demonstrated that mechanical cues delivered by the Vibrating Ingestible BioElectronic Stimulator can activate gastric stretch receptors and trigger systemic physiological responses. Together, these advances affirm the technical feasibility of mechanical sensing in swallowable and compliant devices, though applications for tissue classification remain limited.

Vibration‐based sensing is particularly attractive in this context. Micro‐actuator‐driven oscillations, when coupled with embedded accelerometers, elicit frequency‐dependent responses that reflect local viscoelastic properties [31, 32, 33]. Compared with tactile or pressure‐based techniques, vibrational sensing provides rich spectral features in a compact, energy‐efficient form, well‐suited to miniaturized untethered platforms [34, 35]. Previous demonstrations have shown that vibrational signatures can distinguish tissues of varying stiffness and damping under controlled conditions [36, 37]. However, their translation into ingestible systems capable of reliable in vivo performance is still at an early stage.

The implementation of vibration‐enabled capsules raises several engineering challenges. Excitation frequencies must be matched to sensor sampling rates to avoid aliasing and preserve diagnostic information [38]. Secure coupling between capsule and tissue must be maintained despite peristalsis, fluid flow, and posture changes. In addition, robust processing pipelines are needed to extract discriminative features from noisy, non‐stationary signals. Methods such as Fast Fourier Transform (FFT) [39], machine learning‐based classification [40], and finite element modeling [41] offer promising foundations but require adaptation for real‐time use under dynamic gastrointestinal conditions. These considerations motivate systematic development and validation of vibration‐assisted capsule systems for mechanical tissue characterization.

Colorectal cancer pathology itself further motivates this approach. Carcinogenesis is a multistage process involving genetic mutations, cellular alterations, and disrupted biological pathways, ultimately leading to uncontrolled cell proliferation and tumor formation. While benign tumors remain localized, malignant tumors invade surrounding tissues and metastasize to distant sites such as the liver, lungs, brain, lymph nodes, and peritoneum. In 2022, colorectal cancer accounted for more than 1.9 million new cases and one million deaths globally, making it the third most commonly diagnosed cancer and the second leading cause of cancer mortality [3]. By 2040, incidence is projected to rise to 3.2 million new cases and 1.6 million deaths annually [42]. In England, the 5‐year survival rate is 57.8% [43], but survival varies sharply with stage: 98%, 93%, 89%, and 44% for Stage I–IV diagnosis, respectively [44]. Colonoscopy, the current gold standard, is invasive, costly, and not universally accessible, which contributes to low participation rates and delays in detection. This underscores the urgent need for alternative, less invasive, and widely deployable screening strategies.

A well‐established feature of cancer pathology is the altered mechanics of diseased tissue: cancerous lesions exhibit increased stiffness compared to normal tissue, even at early or sessile stages. Tumor stiffening arises from changes such as increased cell density, extracellular matrix deposition, and interstitial pressure, which are often invisible to conventional imaging. Exploiting this phenomenon, the Exeter Small‐Scale Robotics Laboratory has pioneered the use of millimetre‐scale robots for in situ biomechanical mapping as a new hallmark for colorectal cancer detection [25, 45, 46]. These miniature robots and probes provide gentle, localized measurements of stiffness and viscoelasticity, enabling identification of subtle pathological changes long before they are visible or palpable. Initial studies with vibration‐assisted capsules have demonstrated the ability to detect lesions as small as 2–4 mm, when coupled with AI‐based models [47, 48]. However, earlier prototypes relied on external excitation and off‐board sensors.

In the present study, we report a fully self‐contained vibration‐assisted capsule that integrates the power source, eccentric vibrating mass, and sensing electronics into a single device. The capsule operates autonomously, transmitting triaxial vibrational data wirelessly via Bluetooth to a smartphone for visualization and storage. These data are subsequently processed using an AI‐enhanced classification pipeline to detect and differentiate lesions in ex vivo porcine colon tissue, where artificial tumors of varying stiffness were generated by injected agar mixture. By systematically traversing both lesion and adjacent healthy regions, the capsule demonstrates its capability for real‐time, autonomous biomechanical lesion detection and AI‐supported tissue classification, representing a step forward toward minimally invasive, non‐visual colorectal cancer screening.

## 2 Results

### 2.1 System Overview

**FIGURE 1:**
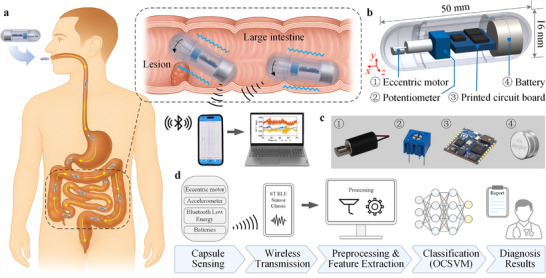
Overview and working principle of the AI‐enhanced vibrational capsule system for bowel sensing and diagnosis. (a) Schematic illustration of the vibrational capsule propelled through the gastrointestinal tract by peristaltic waves. The internal motor generates vibrations that excite the surrounding tissue, while the onboard accelerometer records the corresponding mechanical response. The acquired data are wirelessly transmitted to a smartphone and subsequently processed on a computer for real‐time monitoring. (b) Internal structure of the capsule, consisting of an eccentric motor (①) for vibration excitation, a potentiometer (②) for modulating the capsule's vibration amplitude, a printed circuit board (③) integrating a triaxial accelerometer (x‐, y‐, and z‐axes), a microcontroller, and a communication module, and a battery (④) for power supply. (c) Photographs of the main functional components of the capsule. (d) Data processing workflow: signals from the capsule's accelerometer are wirelessly transmitted via Bluetooth, followed by preprocessing and feature extraction; the processed features are then classified using a One‐Class Support Vector Machine (OCSVM) algorithm to differentiate normal and abnormal tissues. The final diagnostic output can be displayed in a report format for clinical decision support.

The proposed AI‐enhanced vibrational capsule enables untethered, in situ mechanical characterization of intestinal tissues, as illustrated in Figure 1a. After oral ingestion, the capsule is propelled through the gastrointestinal tract by natural peristaltic waves until it reaches the target bowel segment. At this location, an internal eccentric motor generates controlled vibrations that deform the contacting mucosal surface according to its biomechanical properties. A triaxial accelerometer embedded in the onboard electronics records the corresponding acceleration responses, which are wirelessly transmitted via Bluetooth Low Energy (BLE) to a paired smartphone for real‐time visualization and storage.

The structural layout of the capsule is shown in Figure 1b. The capsule has a cylindrical shell (16 mm in diameter, 50 mm in length) housing four integrated modules: ① a miniature eccentric motor for vibration excitation, ② a potentiometer for adjusting the motor drive voltage and thereby modulating vibration amplitude, ③ a commercial printed circuit board (STEVAL‐STLCS01V1) that integrates a triaxial accelerometer, microcontroller unit (MCU), BLE transceiver, and antenna, and ④ two silver‐oxide batteries for power supply. Photographs of the key components are provided in Figure 1c, highlighting their compact arrangement within the capsule enclosure.

The data acquisition and processing workflow is illustrated in Figure 1d. During operation, the eccentric motor excites the surrounding tissue, and the accelerometer captures the resulting vibrational response. Under MCU coordination, the signals are transferred to the BLE module and transmitted wirelessly to a smartphone. Upon acquisition, the triaxial data undergo channel‐wise preprocessing to enhance signal fidelity and remove non‐informative offsets. This includes noise filtering, baseline correction, detrending to suppress slow drift, and de‐meaning (DC removal) to mitigate gravity projection, sensor bias, and low‐frequency baseline drift; accordingly, each channel is mean‐centred by subtracting its sample mean. The de‐meaned signals are then resampled to the nominal device sampling frequency to compensate for intermittent data loss observed during capture or transfer. The uniformly sampled data are partitioned into fixed‐length windows (2 seconds in this study). Each triaxial window is subsequently reduced to a single scalar time series by computing the signal magnitude, thereby consolidating cross‐axis capsule–tissue interactions into a unified representation that captures responses to varying tissue properties. The resulting magnitude signals are transformed into the feature set, which serves as input to the One‐Class Support Vector Machine (OCSVM) for discrimination between normal and abnormal tissues. Prior to model inference, all features are z‐score normalized using statistics computed from the baseline (normal) dataset. Finally, diagnostic outputs are generated in report format to support clinical decision‐making.

### 2.2 Multi‐Channel Signal Differentiation Using a One‐Class Support Vector Machine

The OCSVM, first introduced by Schölkopf et al. [49], learns the function that characterizes the distribution of a single class and constructs a maximum‐margin hyperplane that encompasses normal instances in feature space. This formulation has made OCSVMs widely adopted for anomaly detection, where they effectively handle high‐dimensional data and define clear decision boundaries for outlier detection [50, 51, 52]. Recent applications in rail track inspection [53], sea surface target detection [54], and oil well monitoring [55] demonstrate their utility for real‐time structural and system health monitoring tasks involving subtle and diverse abnormalities.

In this study, the non‐linear OCSVM framework that uses Radial Basis Function (RBF) kernel is adapted for detecting early or hard‐to‐visualize malignant bowel tissues from a background of normal tissues, based on triaxial accelerometer data obtained during ex vivo vibrational capsule experiments. Identifying pathological changes at an early stage is critical for timely intervention in gastrointestinal disease management, particularly bowel cancer, which ranks second in global cancer mortality [3]. Traditional visual endoscopic approaches, while effective, cannot quantify subsurface biomechanical alterations that often precede visual manifestations. Vibration‐assisted capsule endoscopy addresses this gap by acquiring high‐resolution acceleration signals that reflect local tissue mechanical properties as the capsule traverses the gastrointestinal tract [47, 48].

These bio‐signals, however, are inherently non‐stationary due to peristalsis, variable mucosal contact, and active capsule vibrations. In addition, the predominance of healthy tissue in the signal matrix, combined with inter‐patient variability in malignant expression (age, ethnicity, dietary habits), poses challenges for conventional supervised classification. In this context, OCSVM offers an attractive solution by learning only from verified normal bowel segments of an individual patient and then constructs a hyperspace boundary that effectively distinguishes between capsule signatures from healthy and unhealthy tissues. Tissue with mechanical aberrations, such as reduced stiffness or increased compliance typically associated with malignant transformation, manifests as an outlier to this hyperspace and is thus flagged as abnormal. This approach enables early detection without requiring large annotated datasets and supports scalable, patient‐specific screening.

Here, OCSVM is employed to discriminate between normal tissues and three categories of localized malignant tissues (soft, medium, and hard), collectively denoted as abnormal, using multi‐channel acceleration recordings. The raw triaxial signals were consolidated into a single magnitude signal, thereby capturing the capsule's overall interaction with the tissue. These magnitude signals were transformed into feature vectors and used as OCSVM inputs. The model maps these features into a higher‐dimensional space and constructs a hyperplane that separates normal data from the origin, effectively defining a boundary against which deviations are detected [52, 54]. A key strength of OCSVM is its robustness to noise and adaptability to varying data distributions [56, 57], making it particularly suitable for multi‐channel bio‐signals where cross‐channel interactions increase complexity. Recent studies support the benefits of this approach, including differentiation of abnormal oil‐well production [55], fault detection in delta 3D printers [58], and contamination detection in multi‐channel bio‐signals [59]. These examples demonstrate the value of multi‐channel features in enhancing detection accuracy compared to conventional single‐channel methods.

### 2.3 Experimental Design and One‐Class Support Vector Machine Implementation

**FIGURE 2:**
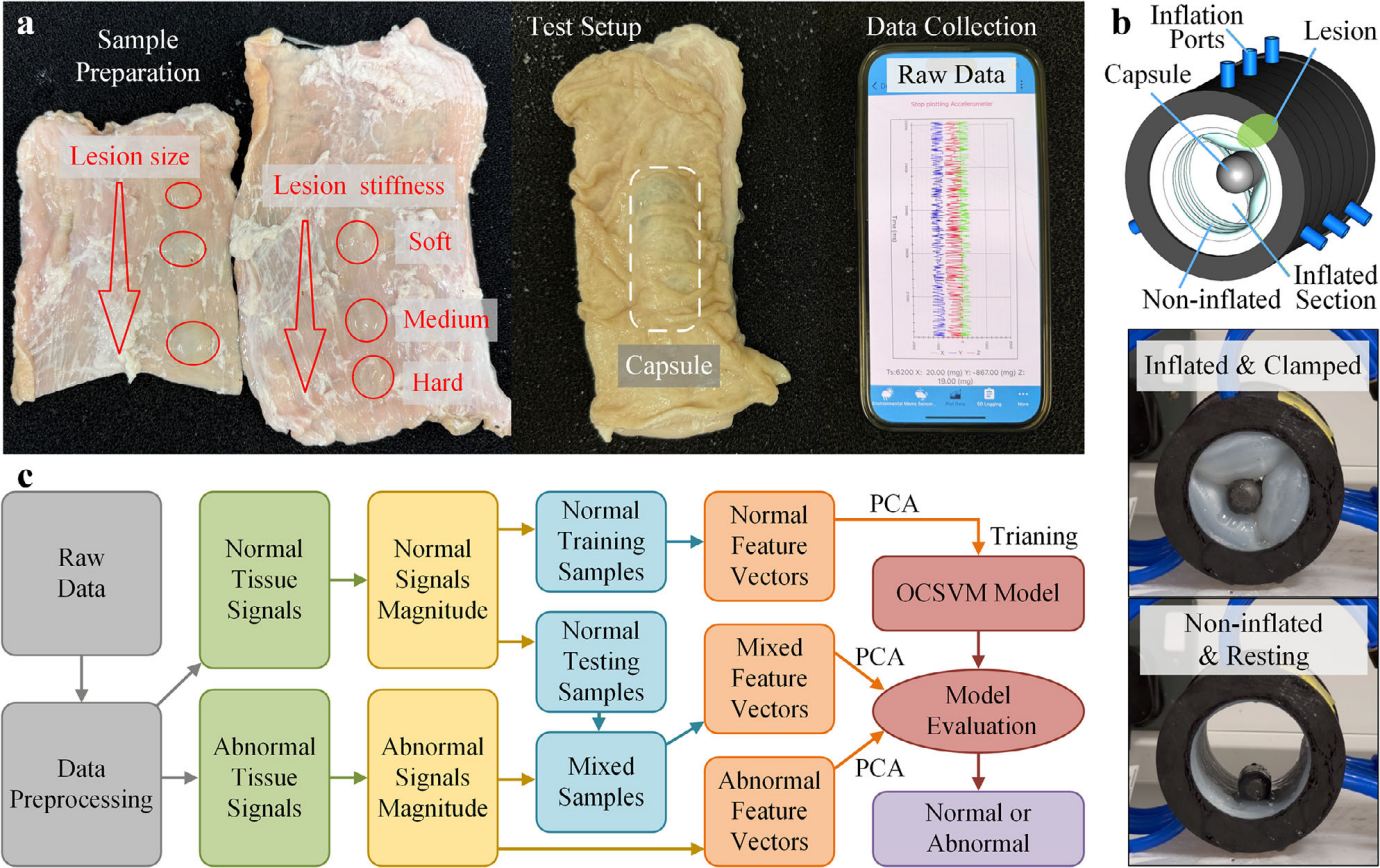
Experimental setups and OCSVM workflow for AI‐enhanced lesion detection. (a) Ex vivo experimental setup for vibration‐based measurement on a porcine colon specimen. Artificial lesions of varying sizes and stiffness levels were created by submucosal agar injection. The capsule, containing a programmable wireless board and eccentric motor, was positioned on the lesion and covered with colon tissue. A smartphone application displayed real‐time acceleration signals transmitted wirelessly from the capsule. (b) Soft pneumatic in vitro colon‐simulator platform used to impose controlled lumen deformation and repeatable capsule‐lesion compressive contact during vibration acquisition. Lesion phantoms with three stiffness levels were mounted on the simulator inner wall, and data were collected under inflated‐clamped and non‐inflated resting configurations. (c) Flow chart of the proposed One‐Class Support Vector Machine (OCSVM) implementation, including preprocessing, feature extraction, feature reduction by principal component analysis (PCA), model training on normal tissue data, then evaluated on abnormal and mixed sets.

To assess the generalizability of the proposed framework and to enhance data diversity across different capsule‐tissue boundary conditions, two complementary experimental platforms were employed. In the bench‐top ex vivo porcine‐colon platform shown in Figure 2a, agar gel was injected submucosally into the porcine colon wall to create stiffness‐increasing abnormalities. In parallel, a soft pneumatic in vitro colon‐simulator platform shown in Figure 2b was employed, where lesion phantoms fabricated from Ecoflex 00‐10, 00‐30, and 00‐50 were attached to a compliant Ecoflex 00‐10 simulator wall to represent soft, medium, and hard tissue conditions, respectively. Controlled pneumatic inflation was applied to modulate lumen geometry and impose repeatable compressive contact at the capsule‐lesion interface.

Signals acquired from both platforms were processed using an identical analysis pipeline as presented in Figure 2c, comprising signal preprocessing, feature extraction, principal component analysis (PCA)‐based dimensionality reduction, and OCSVM‐based training and evaluation. It is important to note that the agar‐based inclusions in the ex vivo porcine‐colon platform and the Ecoflex‐based phantoms in the in vitro simulator were not intended to replicate the absolute stiffness of human neoplastic or fibrotic lesions, although such materials are widely used as tissue surrogates due to their comparable mechanical characteristics [45, 47]. Instead, they provided a controlled means of generating relative stiffness contrasts, enabling systematic evaluation of the capsule's ability to discriminate tissue states based on relative mechanical differences, which underpins the proposed classification approach.

During vibration acquisition in the ex vivo platform, the capsule was gently covered by the colon tissue to ensure consistent contact and confinement. In the in vitro colon simulator, repeatable capsule‐lesion contact was achieved through controlled wall inflation. In both cases, the capsule generated controlled vibrations and recorded the resulting acceleration responses, which were transmitted wirelessly to a smartphone application for real‐time monitoring and data collection.

**FIGURE 3:**
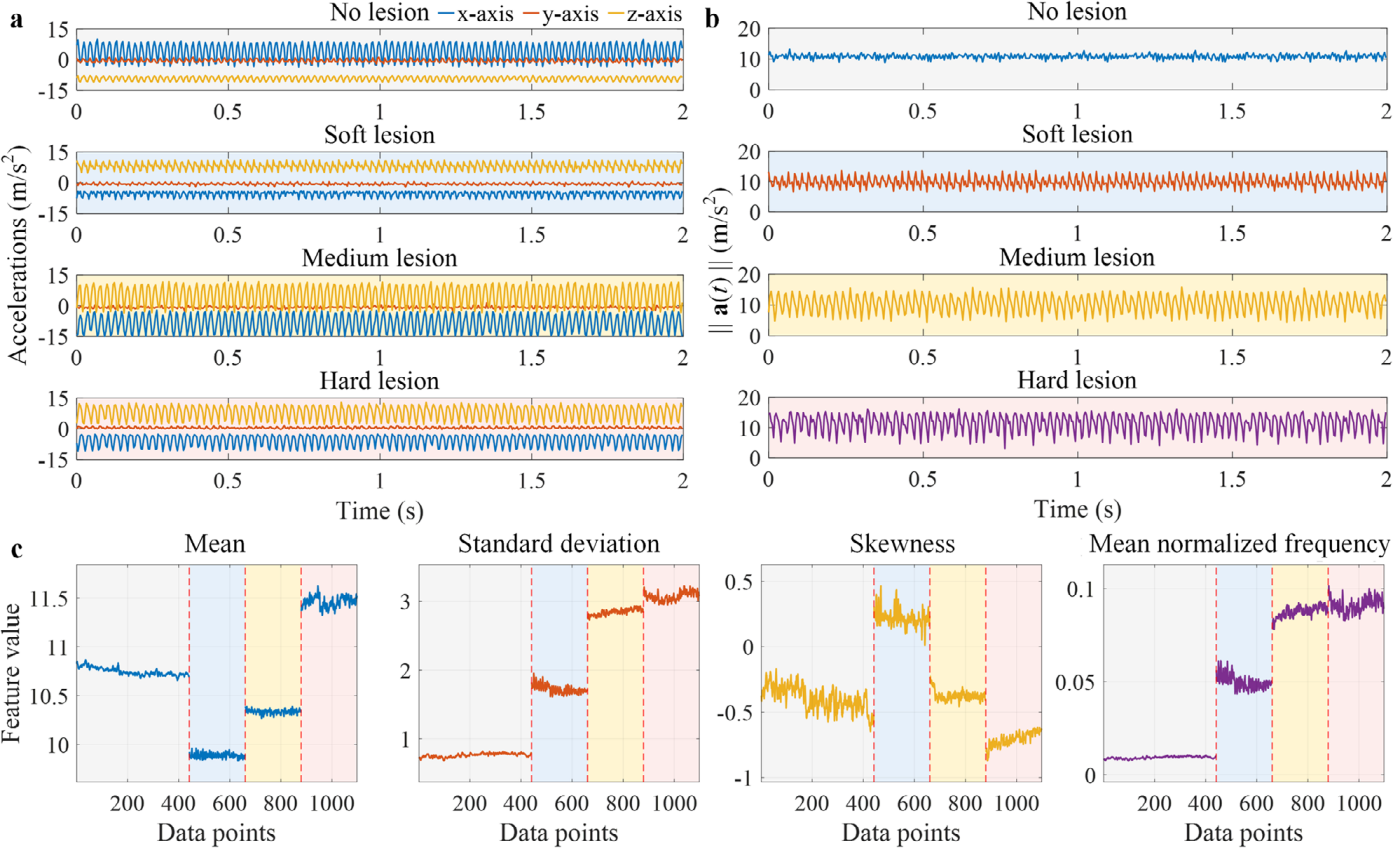
Raw signals, magnitude signals, and feature evolution used for AI‐based tissue classification. (a) Representative triaxial acceleration time histories recorded by the vibrational capsule over four tissue conditions: no lesion, soft lesion, medium lesion, and hard lesion. The three traces correspond to the capsule's x‐, y‐, and z‐axes (blue, red, orange), sampled over a 2s window. Increasing lesion stiffness produces higher‐frequency content and visible waveform distortion relative to the no‐lesion case. (b) Corresponding acceleration magnitude signals ∥a(t)∥ for the same four conditions (no lesion, soft, medium, hard), highlighting overall vibration amplitude and envelope changes independent of sensor orientation. (c) Exemplary feature trajectories computed from tri‐axial signals magnitude spanning the whole dataset including mean, standard deviation, skewness, and mean normalized frequency. Vertical dashed lines mark boundaries between data blocks from different tissue conditions (no lesion, soft, medium, hard). Features such as mean and skewness show step‐like shifts across conditions, while standard deviation and mean normalized frequency increase with stiffness, supporting separability prior to PCA and OCSVM classification.

In total, 441 triaxial signals were recorded over normal tissue regions, and 657 signals (219 for each lesion type) were collected over the soft, medium, and hard lesion conditions using the in vitro platform. For the porcine colon, 1,250 segmented baseline signals were acquired over normal tissue, whereas 4,987 segmented signals were collected in total from agar‐injected sites under varying fluid‐load conditions using the ex vivo platform. Representative examples of the raw triaxial acceleration signals obtained from these regions using the in vitro platform are shown in Figure 3a, while Figure 3c presents their corresponding magnitude signals. The acceleration magnitude was computed from the x‐, y‐, and z‐axes according to

(1) $$∥a\left(t\right)∥=\sqrt{\;{\ddot{x}}^{2}\left(t\right)+{\ddot{y}}^{2}\left(t\right)+{\ddot{z}}^{2}\left(t\right)\;}$$

From these magnitude signals, a total of 37 statistical features, listed in Table 1, spanning the time, frequency, and nonlinear domains were extracted. The resulting feature matrix was mean‐centered, standardized, and subjected to PCA to reduce dimensionality while retaining orthogonal representations for subsequent modeling. Exemplary plots of feature variation across lesion conditions are shown in Figure 3b. Features such as mean and skewness exhibited clear, step‐like shifts between conditions, whereas standard deviation and mean normalized frequency displayed greater overlap, particularly for medium and hard lesions. These results motivated the use of a multi‐feature representation with PCA‐based reduction rather than relying on single descriptors.

**TABLE 1:** Comprehensive list of the 37 dynamic signal features extracted from capsule acceleration data and used for subsequent dimensionality reduction (PCA) and OCSVM‐based classification. The features span three categories: (i) time‐domain descriptors (1–27), quantifying statistical, variability, and shape‐related characteristics of the signals; (ii) frequency‐domain descriptors (28–34), capturing spectral energy distribution and dominant frequency content; and (iii) nonlinear descriptors (35–37), measuring complexity and chaotic properties of the dynamics. Together, these features provide a rich representation of the capsule–tissue interaction, enabling robust discrimination between normal and abnormal tissue responses.

**Time‐domain features**		
1. Mean	2. Minimum	3. Maximum
4. Standard deviation	5. Range	6. Kurtosis
7. Variance	8. Skewness	9. Covariance
10. Mean normalized frequency	11. Avg cumulative maximum	12. Avg cumulative minimum
13. Maximum absolute value‐to‐RMS ratio	14. Root‐mean‐square (RMS)	15. Root‐sum‐of‐squares (RSSq)
16. Crest factor	17. Mean absolute value	18. Form factor
19. Impulse factor	20. Mean square root of absolutes	21. Kurtosis factor
22. Margin factor	23. Skew factor	24. Shape factor
25. Signal‐to‐noise ratio (SNR)	26. SNR‐to‐THD ratio	27. Clearance factor
**Frequency‐domain features**		
28. Mean frequency	29. Median frequency	30. Band power
31. Occupied bandwidth	32. Power bandwidth	33. Peak amplitude
34. Power spectral density		
**Nonlinear features**		
35. Lyapunov exponent	36. Correlation dimension	37. Approximate entropy

The primary aim of this study was to develop an OCSVM model trained exclusively on reduced feature data from normal tissues and apply it to detect deviations from the learned normal manifold. The workflow of this implementation is summarized in Figure 2c. Central to the OCSVM is the choice of kernel *K*, that implicitly defines the feature space and the associated decision function.

As a one‐class method, a Gaussian (radial basis function, RBF) kernel was employed in the current OCSVM and during training it seeks to minimize

(2) $$\frac{1}{2}\sum_{j=1}^{n}\sum_{k=1}^{n}\alpha_{j}\alpha_{k}\;K\left(x_{j},x_{k}\right)$$

with respect to $\alpha_{1},\text{…},\alpha_{n}$, subject to

(3) $$\sum_{j=1}^{n}\alpha_{j}=n\nu ,\;0\leq \alpha_{j}\leq 1,\;\;j=1,\text{…},n$$

where n denotes the number of training samples, K is the kernel function, 0<ν≤1 controls the fraction of support vectors (hence the flexibility of the decision boundary), and $K(x_{j},x_{k})$ represents the Gram matrix entries.

The Gaussian/RBF kernel and the resulting decision function in the form of classification score for an observation x, are respectively given as

(4) $$K\left(x_{j},x_{k}\right)=\exp\;\left(-\gamma ∥x_{j}-x_{k}{∥}^{2}\right),\;\gamma >0$$

and

(5) $$f\left(x\right)=\sum_{j=1}^{n}\alpha_{j}\;K\left(x_{j},x\right)-\rho$$

where $\alpha_{j}\geq 0$ are the learned coefficients and ρ is the offset parameter. Samples with f(x)≥0 (positive score) are treated as inliers indicating conformity to the normal class, while those with f(x)<0 (negative score) are treated as outliers and flag the corresponding observation as anomalous. This formulation enables the model to encapsulate the distribution of normal vibrational signatures, such that deviations induced by abnormal tissue stiffness or compliance are reliably flagged as outliers.

### 2.4 Results of OCSVM‐Based Tissue Classification

The OCSVM‐based classification results are presented in two stages. First, the in vitro platform, operated under controlled contact conditions, is used to establish baseline separability between normal and lesion‐mimicking regions under peristaltic‐like compression representative of colonic loading, with performance quantified using the percentage of inliers (positive) and outliers (negative). Second, the same analysis pipeline is applied to ex vivo porcine‐colon experiments performed under a blinded protocol and across multiple fluid‐load conditions, providing an independent assessment of robustness under more realistic, non‐peristaltic capsule‐tissue boundary conditions.

#### 2.4.1 In Vitro Soft Pneumatic Colon‐Simulator Experiments

During the OCSVM training, an RBF kernel was employed with z‐score standardization using statistics (see Table 1) computed from the baseline dataset. An outlier fraction of ν=0.01 (1%) was specified, allowing a small subset of baseline samples to fall outside the learned decision boundary and consequently receive negative decision scores. The OCSVM, being originally trained on normal data, treats normal samples as inliers and assigns them positive scores, whereas abnormal samples are considered outliers and assigned negative scores. Its performance was summarized in terms of the false‐positive rate (FPR: proportion of abnormal samples [outliers] incorrectly assigned positive scores) and the false‐negative rate (FNR: proportion of normal samples [inliers] incorrectly assigned negative scores).

**FIGURE 4:**
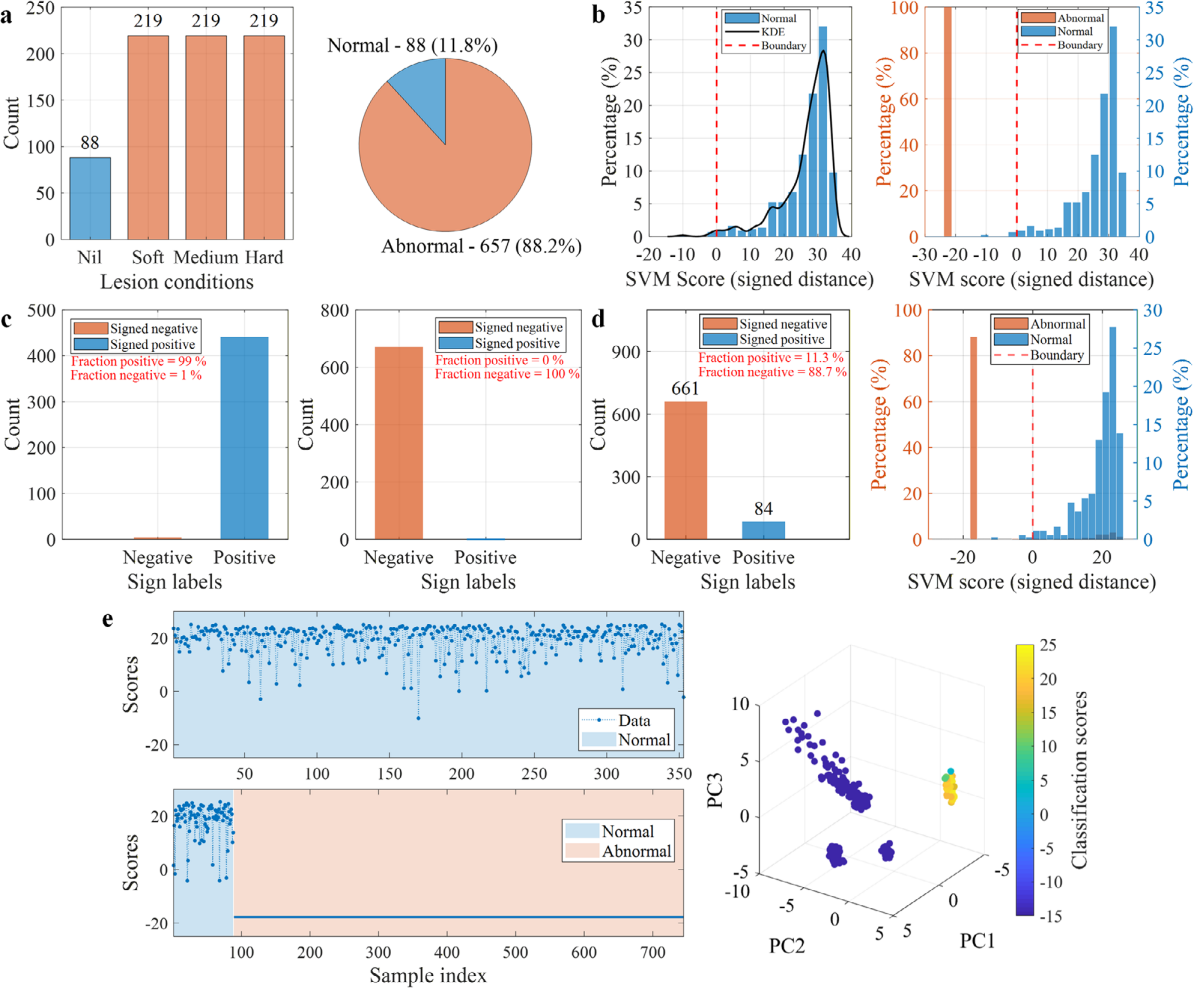
Performance of the OCSVM‐based tissue classification. (a) Composition of the mixed dataset: (left) distribution of lesion categories (nil, soft, medium, hard) and (right) proportion of the normal (11.8%) to the abnormal (88.2%) data samples. (b) Classification score distributions for the Norm‐vs‐Abnorm strategy: (left) training on normal tissue only, showing nearly all samples well above the decision boundary with ∼1% prescribed outliers, and (right) testing on abnormal tissue only, with all the abnormal tissue data correctly classified as outliers. (c) Assigned label counts for the Norm‐vs‐Abnorm strategy during (left) training on normal tissue data and (right) testing on abnormal tissue data, confirming a 0% false‐positive rate. (d) Results for the Norm‐vs‐Mixed strategy: (left) assigned label counts showing four out of 88 normal samples misclassified as abnormal, and (right) classification scores showing clear separation between normal and abnormal data. (e) Classification score analysis for the Norm‐vs‐Mixed strategy: (left top) training on normal tissue only, (left bottom) testing on the mixed dataset, and (right) 3D PCA visualization of the mixed dataset colored by classification scores. The PCA view highlights a compact normal cluster (yellow, high scores) and well‐separated abnormal clusters (blue, low scores).

Figure 4b,c shows the classification scores and assigned labels for the Norm‐vs‐Abnorm strategy. During training, the kernel density estimate of scores indicates that almost all normal samples lie well to the right of the decision boundary, with negligible density below zero, see Figure 4b(left). The small leftward tail corresponds to the 1% outlier allowance, while the sharp positive peak demonstrates that most normal samples are well within the learned boundary. Approximately 1% of normal samples were thus permitted to fall outside the decision region, as prescribed. When evaluated on the abnormal‐only dataset, all samples received negative scores, see Figure 4b(right), giving a true‐negative rate of 100% and a FPR of 0%. Assigned label counts in Figure 4c confirm these outcomes.

As seen from Figure 4a, the Norm‐vs‐Mixed scenario comprised 88 (11.8%) normal and 657 (88.2%) abnormal tissue samples. The classification outcomes are shown in Figure 4d. Of the 88 normal samples, four were misclassified as abnormal, while all abnormal samples were correctly identified. This resulted in an overall accuracy of 99.5%, with a FPR of 0% and a FNR of approximately 4.5% (4/88).

Further insight into the Norm‐vs‐Mixed performance is given in Figure 4e. During training (left top), the normal samples are almost entirely on the positive side of the boundary, apart from the 1% prescribed outliers. In testing (left bottom), the majority of normal samples remain positive, with four dipping below zero, while abnormal samples cluster tightly at large negative scores (around −20). In PCA space (right panel), the normal cluster is compact and associated with high scores (yellow), whereas abnormal clusters are well‐separated and associated with low scores (blue). These results are consistent with the score statistics: TP=84, FN=4, TN=657, FP=0, yielding an overall accuracy of 741/745=99.5%, a normal recall of 84/88=95.5%, and FPR of 0%.

For a like‐for‐like comparison with the present study, only a limited number of works have investigated tactile capsule technologies for mechanical sensing along the gastrointestinal tract; however, these studies employ different hardware platforms, target tasks, and validation geometries, which precludes direct benchmarking. For example, Camboni et al. [60] developed an endoscopic tactile robotic capsule embedded with miniaturized MEMS force sensors for hardness‐based detection of non‐polypoid colorectal tumors, reporting an accuracy of 94%. Similarly, Peker et al. [61] used a force‐sensing capsule to quantify tissue stiffness and estimated Young's modulus values of 11.3±2.3kPa for healthy tissue and 26.8±4.6kPa for cancerous tissue. Ge et al. [62] also utilized tactile sensors to measure tissue‐capsule interaction forces/pressure for detecting early‐stage small intestinal nodules.

#### 2.4.2 Ex Vivo Porcine Colon Experiments

The above initial experiment was designed to establish the feasibility of the proposed method under controlled conditions and well‐differentiated tissues including soft, medium and hard. However, we acknowledge that the lack of explicit blinding at this stage means the reported performance could be susceptible to unintentional bias. To ensure rigorous independence between specimen preparation and measurement and analysis, which is particularly important for localized tissue interrogation coupled with data‐driven classification, we implemented a blinded experimental protocol with strict separation of roles across lesion preparation, data acquisition, and machine learning based categorization.

In this revised protocol, the first operator prepared the porcine colon specimen and pre‐marked multiple points on it for capsule data acquisition. While the specimen remained intact and prior to any agar injections, a second operator acquired vibration signals at all marked sites to establish baseline pre‐injection reference data for normal tissue. The first operator then injected agar at a randomly selected subset of the marked sites and took no further part in capsule placement, data collection, or analysis. Crucially, the second operator, blinded to both lesion presence and lesion location, subsequently repeated sequential capsule placement at all marked sites and re‐acquired vibration data under identical experimental conditions, without any visual or tactile cues indicating lesion status. Different agar mixtures were prepared and used for injection to introduce variation. However, the precise stiffness change induced in the tissue by each mixture was not controlled precisely as in the first experiment. To improve statistical robustness and better mimic in vivo intraluminal conditions, signals were acquired in triplicate at every marked site and under different fluid loaded environments, including saline and a chyme mimicking lubricant.

For analysis, the machine learning modeller received two datasets. The first comprised baseline pre‐injection signals comprising normal tissue signals. The second comprised post‐injection data containing a mixture of normal and agar injected tissues signals. Data processing and AI classification were performed independently based solely on signal characteristics, without access to lesion labels during modeling and the lesion ground truth was only revealed after data classification pipeline, enabling an unbiased evaluation of the proposed sensing method.

**FIGURE 5:**
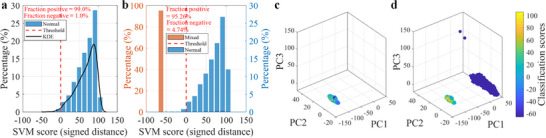
OCSVM evaluation on baseline and blinded mixed datasets using decision‐score distributions and PCA embeddings. (a) Histogram of OCSVM decision scores (signed distance) for the baseline (normal) training dataset, including the threshold (red dashed line) and kernel density estimate (black line), showing that 99.0% of baseline signatures are retained as normal. (b) Score distributions for the blinded mixed dataset (orange; left axis) overlaid with the baseline score distribution (blue; right axis), illustrating that 95.26% of blinded signatures are classified as abnormal (negative) and 4.74% as normal (positive) relative to the same threshold. (c) PCA feature‐space embedding of the baseline dataset colored by OCSVM decision scores, forming a single compact high‐score cluster. (d) PCA embedding of the blinded mixed dataset colored by OCSVM decision scores, showing two distinct compact clusters, a higher‐score cluster overlapping the baseline region and a separate lower‐score cluster corresponding to agar‐injected (adenoma‐like) tissues.

Figure 5 summarizes the behavior of the OCSVM under training and blinded evaluation. In the left panel Figure 5a, the OCSVM is trained exclusively on baseline signatures acquired from normal tissues, with the outlier fraction set to ν=0.01 (i.e., a 1% contamination level). The learned decision boundary tightly encloses the dominant normal‐data distribution, as reflected by the predominantly positive signed distances. Quantitatively, 99.0% of the normal training samples are retained as inliers, while 1.0% are rejected as outliers, confirming that the chosen ν enforces the intended tolerance whilst preserving the baseline manifold. In the right panel Figure 5b, the trained model is evaluated on a blinded mixed dataset comprising the baseline (normal) signatures and the agar‐injected (abnormal) tissue signatures. The abnormal signatures produce strongly negative signed distances (orange) and cluster well below the learned threshold, indicating robust outlier detection. Notably, 4,989 (95.26%) of the blinded signatures were classified as negative (abnormal), while 248 (4.74%) were retained as positive (normal), appearing as a faint shadow beneath the baseline categories (blue bar). Following clarification with the operator who prepared the samples, it was confirmed that the blinded dataset comprised 250 baseline (normal) tissue signatures and 4,987 abnormal tissue signatures that were collected under void, saline, and chyme‐mimicking fluid‐load conditions. Accordingly, this implies an FNR of 0.8% (2/250).

The distribution of the baseline dataset and the mixed blinded dataset in the utilized PCA feature space, colored by the OCSVM decision scores, is shown in Figure 5c,d, respectively. The baseline (normal) dataset presented in Figure 5c forms a single compact cluster in which the signatures group tightly and exhibit predominantly higher scores (warm colors). By contrast, the mixed blinded dataset presented in Figure 5d shows two distinct compact clusters, one overlaps the baseline region and retains higher scores that are consistent with the learned normality manifold, thus indicating the normal tissue signatures embedded within the blinded set. The second cluster occupies a separate region and is characterized by uniformly low (more negative) scores (cool colors), corresponding to the agar‐injected (adenoma‐like) tissues. This strong score contrast and clear geometric separation indicate robust discrimination of abnormal biomechanical perturbations relative to baseline. Notably, the normal signatures exhibit wider variation in the PCA feature space than the abnormal cluster. In addition, within the blinded dataset it was not possible to differentiate between the different agar stiffness conditions under the various fluid‐load conditions.

Overall, these findings indicate that an OCSVM trained solely on normal porcine colon tissue signatures generalizes effectively to unseen data by capturing the baseline distribution, preserving embedded baseline components within the blinded mixed set, and achieving robust separation between normal and abnormal signals with consistently low false‐positive rates and reliable detection of lesion‐induced biomechanical perturbations. Therefore, the results underscore the practicality of coupling vibrational capsule measurements with OCSVM‐based analysis for early, non‐visual identification of malignant transformations, providing a promising framework for minimally invasive, real‐time mucosal screening that complements conventional imaging modalities in bowel cancer detection.

### 2.5 Validation of AI Classification via Dynamic Modeling

**FIGURE 6:**
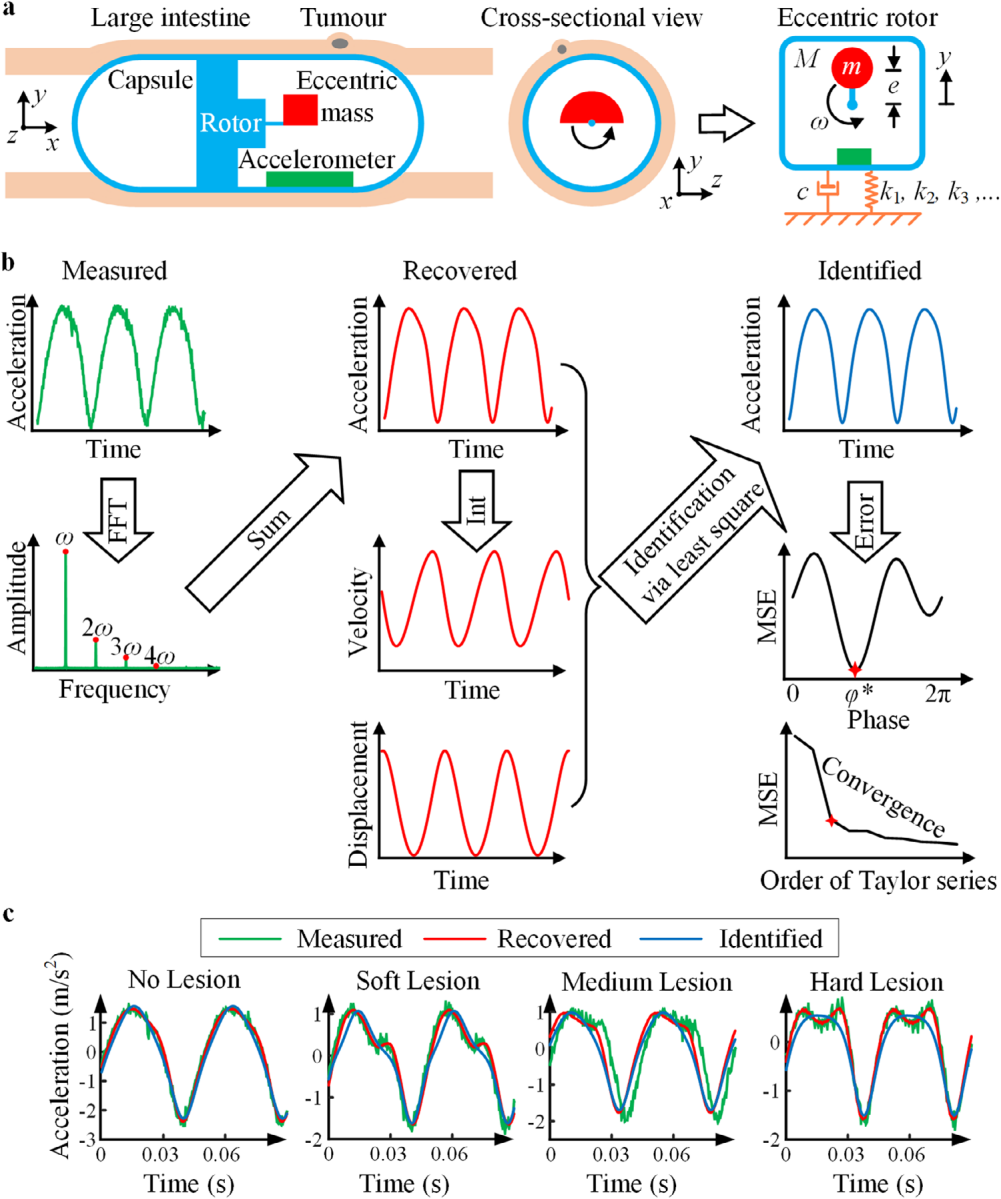
Dynamic modeling and parameter identification of capsule‐tissue interaction. (a) Equivalent model of the vibrational capsule. The capsule excites vertical oscillations by rotating an eccentric mass, generating a periodic inertial force. The surrounding intestinal tissue constrains motion through damping (c) and nonlinear stiffness terms ($k_{1},k_{2},k_{3},\text{…}$). A cross‐sectional view illustrates the eccentric rotor and embedded accelerometer used for sensing. (b) Schematic of the proposed identification method. The measured acceleration signal is first transformed into the frequency domain using Fast Fourier transform (FFT) to identify the dominant frequency and harmonics. The signal is then reconstructed by summing harmonic components, from which velocity and displacement are obtained by integration. A least‐squares procedure is applied to match reconstructed dynamics with the governing model, while iteratively varying the excitation phase ϕ to minimize mean square error (MSE). The convergence of MSE with increasing Taylor series order determines the necessary level of nonlinearity in the stiffness representation. (c) Comparison of measured (green), recovered (red), and identified (blue) acceleration responses for four experimental cases: no lesion, soft lesion, medium lesion, and hard lesion. The no‐lesion case exhibits a nearly sinusoidal response consistent with linear behavior, whereas lesion‐contact cases show visibly distorted waveforms due to nonlinear stiffness effects. The close agreement between measured and identified signals demonstrates the accuracy of the identification method.

To further validate the proposed AI‐based classification, an equivalent dynamic model of the vibrational capsule interacting with colon tissue was developed, as shown in Figure 6a. The capsule is driven by an eccentric rotor motor, which generates a periodic inertial force,

(6) $$F\left(t\right)=me\omega^{2}\sin\left(\omega t+\phi \right)$$

where m is the eccentric mass, e the eccentricity, and ω and ϕ are the excitation frequency and phase. The vertical motion of the capsule is governed by

(7) $$M\ddot{y}+c\dot{y}+f\left(y\right)=F\left(t\right)$$

where M is the capsule mass, y the vertical displacement, c the damping coefficient, and f(y) the nonlinear restoring force from the surrounding tissue. For small‐amplitude oscillations, the restoring force is approximated by a Taylor expansion:

(8) $$f\left(y\right)\approx \sum_{i=1}^{N}k_{i}y^{i}$$

where $k_{i}$ are equivalent stiffness coefficients.

Before experiments, the capsule mass M, eccentric mass m, and eccentricity e are determined from geometry and material properties. During experiments, the capsule acceleration $\hat{\ddot{y}}\left(t\right)$ is measured by the onboard accelerometer. The task is then to identify parameters ω, ϕ, c, and $k_{i}$ (i=1,2,⋯,N) from known values of M,m,e, and the measured acceleration $\hat{\ddot{y}}\left(t\right)$.

The identification procedure is illustrated in Figure 6b and summarized as follows. From $\hat{\ddot{y}}\left(t\right)$, FFT yields the dominant frequency ω. The presence of higher harmonics (2ω,3ω,⋯) indicates nonlinearities in capsule‐tissue interaction, so a purely linear model is insufficient. To reduce noise, the measured acceleration is reconstructed by summing harmonics

(9) $$\hat{\ddot{y}}\left(t\right)\approx \bar{\ddot{y}}\left(t\right)=\sum_{j=1}^{H}\left[a_{j}\sin\left(j\omega t\right)+b_{j}\cos\left(j\omega t\right)\right]$$

where $a_{j},b_{j}$ are Fourier coefficients and H is the highest harmonic above noise. By integration, velocity and displacement are recovered as

(10) $$\begin{array}{cc} \bar{\dot{y}}\left(t\right)= & \sum_{j=1}^{H}\left(-\frac{a_{j}}{j\omega }\cos\left(j\omega t\right)+\frac{b_{j}}{j\omega }\sin\left(j\omega t\right)\right)\\ \bar{y}\left(t\right)= & \sum_{j=1}^{H}\left(-\frac{a_{j}}{{\left(j\omega \right)}^{2}}\sin\left(j\omega t\right)-\frac{b_{j}}{{\left(j\omega \right)}^{2}}\cos\left(j\omega t\right)\right) \end{array}$$

The excitation force can then be expressed as

(11) $$F\left(t\right)=\bar{F}\left(t\right)=me\omega^{2}\sin\left(\omega t+\bar{\phi }\right),$$

with phase $\bar{\phi }$ initially arbitrary. Substituting Equations (8)–(11) into Equation (7) gives

(12) $$c\bar{\dot{y}}\left(t\right)+\sum_{i=1}^{N}k_{i}{\bar{y}}^{i}\left(t\right)=\bar{F}\left(t\right)-M\bar{\ddot{y}}\left(t\right)$$

Discretizing time with step Δt yields a linear system

(13) AX=b

where

(14) $$\begin{array}{ccc} & & A=\left(\begin{array}{ccccc} \bar{\dot{y}}\left(\Delta t\right) & \bar{y}\left(\Delta t\right) & {\bar{y}}^{2}\left(\Delta t\right) & \cdots & {\bar{y}}^{N}\left(\Delta t\right)\\ \bar{\dot{y}}\left(2\Delta t\right) & \bar{y}\left(2\Delta t\right) & {\bar{y}}^{2}\left(2\Delta t\right) & \cdots & {\bar{y}}^{N}\left(2\Delta t\right)\\ \vdots & \vdots & \vdots & \ddots & \vdots \\ \bar{\dot{y}}\left(T\Delta t\right) & \bar{y}\left(T\Delta t\right) & {\bar{y}}^{2}\left(T\Delta t\right) & \cdots & {\bar{y}}^{N}\left(T\Delta t\right) \end{array}\right),\\ & & b=\left(\begin{array}{c} \bar{F}\left(\Delta t\right)-M\bar{\ddot{y}}\left(\Delta t\right)\\ \bar{F}\left(2\Delta t\right)-M\bar{\ddot{y}}\left(2\Delta t\right)\\ \vdots \\ \bar{F}\left(T\Delta t\right)-M\bar{\ddot{y}}\left(T\Delta t\right) \end{array}\right) \end{array}$$

The unknown parameters are contained in

(15) $$X=\left(\begin{array}{c} c\\ k_{1}\\ k_{2}\\ \vdots \\ k_{N} \end{array}\right)$$

and estimated via least squares

(16) $$X^{*}=\left(\begin{array}{c} c^{*}\\ k_{1}^{*}\\ k_{2}^{*}\\ \vdots \\ k_{N}^{*} \end{array}\right)={\left(A^{T}A\right)}^{-1}A^{T}b$$

The method of least squares is sufficient for many cases, but noise in the measurement could result in physically impossible parameters, such as a negative damping. In that case, we use standard quadratic programming to obtain $X^{*}$ by minimizing ${\left(AX-b\right)}^{T}\left(AX-b\right)$ subjected to c≥0.

Since the excitation phase ϕ is unknown, the procedure is repeated across discrete values of ϕ∈[0,2π). For each trial phase, reconstructed acceleration

(17) $${\ddot{y}}^{*}\left(i\Delta t\right)=\frac{1}{M}\left(\bar{F}\left(i\Delta t\right)-c^{*}\bar{\dot{y}}\left(i\Delta t\right)-\sum_{L=1}^{N}k_{L}^{*}{\bar{y}}^{L}\left(i\Delta t\right)\right)$$

is compared with measured acceleration $\hat{\ddot{y}}\left(i\Delta t\right)$. The mean square error (MSE) is

(18) $$\text{MSE}=\frac{1}{T}\sum_{i=1}^{T}{\left({\ddot{y}}^{*}\left(i\Delta t\right)-\hat{\ddot{y}}\left(i\Delta t\right)\right)}^{2}$$

The optimal phase $\phi^{*}$ minimizes the MSE, and the corresponding parameter vector $X^{*}$ is selected.

By repeating this procedure for increasing Taylor series order N, the convergence of MSE indicated the required degree of nonlinearity. Parameters identified using the analytical method are summarized in Table 2, with corresponding signal comparisons shown in Figure 6c. The results reveal that the damping coefficient c is negligible, remaining close to zero across all cases. In contrast, the linear stiffness $k_{1}$ increases monotonically with lesion stiffness, confirming that stiffer lesions yield higher equivalent stiffness values. Furthermore, nonlinear contributions become increasingly significant when the capsule interacts with lesions: the quadratic ($k_{2}$) and cubic ($k_{3}$) stiffness terms for soft, medium, and hard cases are substantially larger than in the no‐contact condition. This behavior is consistent with the waveform characteristics in Figure 6c, where the no‐contact response resembles a simple sinusoid, while contact with lesions produces clearly distorted signals indicative of nonlinear effects. Together, these findings demonstrate that the identified stiffness coefficients not only capture lesion stiffness but also validate the AI‐based classification framework by providing a physical interpretation of the observed signal distinctions.

**TABLE 2:** Identified parameters for four different samples, where c, $k_{1}$, $k_{2}$, $k_{3}$ and $R^{2}$ are damping coefficient, linear stiffness, quadratic stiffness, cubic stiffness, and the coefficient of determination, respectively.

Sample	c [$\text{Ns}\;m^{-1}$]	$k_{1}$ [$N\;m^{-1}$]	$k_{2}$ [$N\;m^{=2}$]	$k_{3}$ [$N\;m^{-3}$]	$R^{2}$
No Lesion	0.02	225.06	$1.83\times 10^{5}$	$0.50\times 10^{10}$	0.9698
Soft Lesion	0	225.78	$2.39\times 10^{5}$	$3.57\times 10^{10}$	0.9283
Medium Lesion	0	311.92	$5.03\times 10^{5}$	$1.49\times 10^{10}$	0.8617
Hard Lesion	0.02	368.35	$11.80\times 10^{5}$	$2.76\times 10^{10}$	0.8855

### 2.6 Discussion

The results demonstrate that vibration‐assisted capsule sensing, when coupled with OCSVM analysis, provides a reliable means of distinguishing biomechanical differences between normal and abnormal colonic tissue. The measured acceleration responses exhibit systematic variations that reflect lesion‐induced changes in tissue stiffness and viscoelastic behavior. These experimental observations are further supported by the analytical model, which identifies equivalent stiffness coefficients that increase monotonically with lesion stiffness, alongside increasingly prominent nonlinear terms when the capsule interacts with lesion regions. Thus, the experimental and modeling results confirm that vibrational response features encode meaningful mechanical information and can be exploited for robust lesion detection.

Despite these promising findings, several limitations remain. First, the current validation was restricted to ex vivo porcine colon specimens, which do not fully replicate the dynamic environment of the living gastrointestinal tract. In the present study, data acquisition was performed under controlled boundary conditions to ensure stable capsule‐tissue contact and measurement repeatability, which inevitably differs from freely resting in vivo states. In particular, the agar‐based inclusions employed in this study were not intended as clinical surrogates of colorectal lesions, but as controllable and reproducible mechanical benchmarks for validating stiffness‐ and viscoelasticity‐sensitive sensing performance. This approach is consistent with established practice in elastography, mechanical palpation, and robotic tissue sensing, where artificial inclusions are widely adopted as an intermediate validation step prior to evaluation on biological lesions or in vivo models [63, 64].

Peristalsis, mucosal fluid layers, and temperature variations may introduce additional complexities not captured in this study. However, since colonic peristalsis generally occurs at a very slow rate (on the order of 2–4 contractions per day), the capsule can reasonably be considered to operate under quasi‐static conditions during localized in vivo measurements, mitigating some of the potential influence of peristaltic motion on vibrational data. While local segmental motions and transient disturbances may still occur in vivo, the relatively short acquisition window and localized nature of the measurements suggest that their influence on the extracted vibration features is limited. In addition, tissue hydration and temperature can modulate viscoelastic properties and contact mechanics, and although the specimens were continuously hydrated during ex vivo testing to preserve elasticity, hydration gradients, surface wetness, and dehydration over time may still introduce variability in the measured vibration signatures; likewise, the in vivo temperature (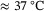) and patient‐specific bowel motility (e.g., transient segmental contractions) may alter capsule‐tissue coupling conditions and thereby affect vibration responses. Moreover, external noise sources, such as mechanical vibrations from handling or electronic interference, may influence signal fidelity, while variability in boundary conditions (e.g., degree of tissue coupling) can affect repeatability. Importantly, because the measurements reflect the equivalent stiffness of the tissue in direct contact with the capsule, they are not sensitive to capsule orientation. While strict alignment was enforced in the present experiments to ensure reproducibility, orientation is expected to be less critical in practice, as the analysis relies on the magnitude of triaxial acceleration, which is inherently invariant to rotation. This suggests a degree of robustness under realistic in vivo conditions, although further validation will be required. Finally, the present implementation relies on offline processing, which is sufficient for validation but limits immediate real‐time deployment.

From a translational point of view, the present prototype is intended as a controlled ex vivo validation platform rather than a clinically deployable system. While the current results demonstrate the feasibility of vibration‐based biomechanical sensing under well‐defined boundary conditions, further system‐level optimization, such as reducing its dimension and maximizing its power, will be required to accommodate the variability of the gastrointestinal environment. To better define clinical applicability and integration into prevailing colorectal cancer screening pathways, the proposed vibration‐assisted sensing capsule is intended as a complementary biomechanical modality rather than a replacement for colonoscopy. In the near term, two realistic roles can be envisaged: (i) early triage or risk stratification, where segments exhibiting abnormal mechanical signatures could prompt prioritization of confirmatory investigations, and (ii) adjunct detection alongside imaging‐based workflows (e.g., capsule endoscopy or colonoscopy), where vibration‐derived mechanical cues can provide non‐visual evidence of abnormal tissue properties and flag regions for targeted inspection and biopsy. In addition, practical deployment will need to consider patient tolerability and motion‐related artifacts. Although the present study operates in a localized, low‐amplitude excitation regime for sensing, comprehensive in vivo evaluation will be required to quantify comfort and safety (e.g., packaging robustness, thermal characteristics, and biocompatibility). Furthermore, intermittent tissue contact, mucosal fluid layers, and transient segmental motions may introduce motion artifacts and variability in coupling conditions. While the short acquisition window and the use of rotation‐invariant triaxial acceleration magnitude features mitigate sensitivity to capsule orientation, additional validation under physiological conditions will be necessary. Finally, any path toward clinical translation will require staged preclinical verification and regulatory planning that may be aligned with a Class II medical‐device pathway (e.g., risk management, verification/validation, and clinical evidence generation).

## 3 Conclusion

This work has presented the development and validation of a vibration‐assisted capsule system for minimally invasive detection of abnormal colonic tissue. The capsule integrates a self‐contained vibration source, triaxial accelerometer, wireless transmission, and power supply, enabling untethered acquisition of biomechanical signals in situ. The experimental framework was complemented by theoretical modeling of capsule–tissue dynamics and analytical identification of stiffness coefficients, which confirmed consistent trends between lesion stiffness and measured vibrational responses. On this basis, an AI pipeline using an OCSVM successfully discriminated normal from abnormal tissue signatures, achieving high accuracy with a negligible false‐positive rate.

These findings indicate that vibration‐based sensing is a viable modality for lesion detection in the colon. By capturing mechanical properties such as stiffness and nonlinear compliance, the capsule enables classification of subtle tissue abnormalities that are difficult to visualize by conventional imaging alone. This study, therefore, establishes a foundation for real‐time, non‐visual diagnostics using a wireless, low‐power capsule platform.

Future work will focus on system‐level miniaturization and higher integration of onboard electronics, actuator, and power architectures, which are necessary steps toward reducing the capsule dimensions for eventual in vivo and translational studies. In parallel, validation in large‐animal models will be required to evaluate robustness under physiological conditions and to further refine both hardware and algorithms toward clinical reliability. In addition, future studies will explore multi‐frequency excitation protocols to improve sensitivity and robustness (leveraging the existing capability to modulate excitation frequency across a stable operating range), investigate deep‐learning‐based models that directly learn robust end‐to‐end representations from raw triaxial waveforms, and expand datasets toward human tissues and clinically relevant pathologies to establish generalizability under realistic physiological variability. Finally, future developments could also focus on advancing toward intelligent biopsy capsule systems, with particular emphasis on system‐level miniaturization and higher integration of onboard electronics, actuation, and power architectures. These developments are expected to enable more compact and clinically deployable implementations while supporting advanced functionalities, such as onboard classification, targeted sampling, and localization.

## 4 Experimental Section

### 4.1 Capsule Fabrication and Modulation

The capsule prototype was designed in SolidWorks and fabricated via fused deposition modeling 3D printing using polylactic acid. The internal system comprises four modules: an eccentric motor, a potentiometer‐based voltage regulator, a coin‐cell power source, and an onboard sensing and communication unit. These components were compactly assembled into a cylindrical housing, producing a device suitable for experimental deployment.

The sensing and communication module (STEVAL‐STLCS01V1, STMicroelectronics) integrates a triaxial accelerometer and a BLE transmitter, enabling wireless acquisition and real‐time transmission of acceleration data during ex vivo testing. Power was provided by two silver oxide coin cells (Model: 386‐301TZ, Energizer) connected in series, supplying a nominal voltage of 3.1 V and a total capacity of 127 mAh. This configuration ensured sufficient energy for uninterrupted operation during extended trials, eliminating the need for battery replacement or recharging. The eccentric motor (VZ4TL2B0620044P, Vybronics) was embedded in the capsule not for propulsion, but as a localized mechanical excitation source, generating vibrations to probe tissue–capsule interactions.

According to the motor datasheet, the rated rotational speed is 8,000 rpm at 3.0 V, corresponding to a vibration frequency of approximately 133 Hz. This exceeds the Nyquist limit of the onboard triaxial accelerometer, which supports a maximum stable sampling rate of 200 Hz (corresponding to a Nyquist frequency of 100 Hz). Operating the motor at full voltage would therefore induce aliasing and amplitude distortion. To prevent this, the motor was driven at reduced voltages to maintain excitation frequencies within the accelerometer's effective bandwidth. A voltage‐frequency calibration was established using high‐speed videography. Rotational dynamics were captured with a Kron Technologies Chronos 2.1 high‐speed camera, with a pre‐marked indicator affixed to the motor shaft. Frame‐by‐frame tracking of angular displacement enabled the extraction of vibration frequencies across the tested voltage range. Stable operation was observed for voltages between 0.6 and 1.5 V, yielding frequencies from 17 to 63 Hz, safely below the Nyquist threshold.

At the selected operating voltage of 1.0 V, the resulting vibration frequency (∼47 Hz) lies well within the effective bandwidth of the onboard triaxial accelerometer, ensuring reliable capture of vibration‐induced tissue responses without aliasing. The vibration amplitude was indirectly controlled via the motor drive voltage and maintained within a low‐amplitude regime suitable for localized mechanical excitation rather than propulsion. Under these operating conditions, the overall power consumption remained sufficiently low to enable continuous operation over extended experimental durations using coin‐cell batteries, while maintaining stable BLE data transmission without observable packet loss during ex vivo testing.

To enable fine control of excitation, a miniature rotary potentiometer (3362P‐1‐501LF, Bourns) was connected in series between the power supply and the motor. Manual adjustment of the potentiometer resistance permitted precise tuning of the motor input voltage, allowing modulation of vibration frequency. This control was essential to maintain excitation frequencies within the accelerometer bandwidth, reduce aliasing, and ensure stable vibrational output during soft tissue interaction experiments.

### 4.2 Experimental Setup and Procedure

Porcine colon specimens for the ex vivo testing were selected from thick, uniform segments to minimize variations in wall thickness and ensure experimental consistency. Experiments were conducted on multiple anatomically distinct pieces of ex vivo porcine colon, harvested from different regions and prepared independently. Each colon segment was treated as an independent specimen with its own baseline mechanical characteristics, and multiple predefined measurement locations (typically 12–20 per segment, depending on tissue size) were marked to capture intra‐specimen mechanical variability.

Each specimen was thoroughly cleaned of residual contents, continuously hydrated to preserve elasticity, and longitudinally cut open to expose the mucosal surface. Artificial inclusions were generated by submucosal injection of agar solutions at predefined locations, as illustrated in Figure 2a. Mixed agar solutions were prepared at concentrations of 0.5%, 1%, and 2% (powder weight to water) by dissolving agar powder in water within glass beakers, heating to 100 °C and mixing until fully dissolved. The liquid agar mixture was injected into the submucosal layer using blunt‐tip dispensing needles attached to syringes, with approximate injection volumes of 1, 2, and 3 ml corresponding to small, medium, and large inclusions, respectively. Following injection, the inclusions were gently shaped and allowed to set for 40 mins at room temperature, forming stable, well‐defined benchmarks. These agar‐based inclusions served as controllable mechanical benchmarks for systematic validation of the proposed vibration sensing and classification framework, rather than as clinical surrogates of colorectal lesions [65, 66, 67].

For the ex vivo measurements shown in Figure 2a, the cut‐open colon specimen was mounted mucosa‐side up on a compliant foam base. During vibration acquisition, the capsule was placed on the mucosal surface over the inclusion site and gently covered by the colon‐tissue layer to maintain consistent confinement and capsule‐tissue contact. Two lesion‐variation conditions were investigated: (i) varying inclusion size at a fixed agar concentration, and (ii) varying inclusion stiffness by changing agar concentration at a fixed injection volume. Lesions were grouped into three representative categories (soft, medium, and hard) for subsequent analysis.

Ecoflex‐based silicone elastomers were selected for the soft pneumatic colon simulator due to their widespread use in soft robotics and tissue‐mimicking phantoms, with well‐characterized compliant and viscoelastic behavior under dynamic loading [68, 69, 70]. The simulator body (colon wall) was fabricated from Ecoflex 00–10 and pneumatically actuated to impose repeatable lumen deformation and controlled capsule‐lesion compressive contact. Lesion phantoms with three stiffness levels were fabricated from Ecoflex 00–10, 00–30, and 00–50 and fixed on the simulator inner wall at a predefined lesion location. For primary data acquisition used in model training and evaluation, the simulator was operated in an inflated and mechanically clamped configuration. Inflation was applied through three circumferentially distributed groups of inlet ports to promote a more uniform pressure field around the lumen, and the fixture was subsequently clamped to stabilize the geometry and minimize motion at the test region. A non‐inflated resting configuration was additionally recorded for qualitative comparison.

For each trial of the in vitro measurements shown in Figure 2b, the capsule was aligned to a consistent position relative to the lesion site to ensure repeatability of accelerometer axes across repeated measurements. The eccentric motor was then activated to generate vibrations, and 20 s of continuous acceleration data were recorded at a sampling rate of 200 Hz. Each configuration was tested in triplicate to evaluate repeatability. The recorded acceleration signals were subsequently analyzed to quantify the influence of lesion stiffness and size on vibrational response characteristics.

#### 4.2.1 Intraluminal Fluid Conditions

Native intestinal chyme was compositionally complex and highly variable, whereas gastrointestinal mucus behaves as a predominantly aqueous, viscoelastic gel that serves a primary lubricating function at the tissue‐lumen interface. Prior studies have shown that mucus‐like or biosimilar hydrogels can reproduce essential rheological and lubrication characteristics of native intestinal mucus under soft‐contact conditions [71, 72, 73]. Accordingly, a saline‐based lubricating medium was adopted as a practical and reproducible approximation of intraluminal fluid loading.

Ex vivo porcine‐colon experiments were conducted under fluid‐loaded conditions. Specifically, the colonic lumen was filled with (i) a 0.9% saline solution and (ii) a saline‐based, mucus‐chyme‐mimicking medium prepared by mixing saline with a commercial water‐based lubricating gel. The saline‐lubricant mixture was prepared using a fixed volumetric mixing ratio and applied consistently across all experiments to ensure repeatability. The saline‐lubricant mixture introduced increased viscosity and lubrication at the capsule‐tissue interface, resulting in additional mass loading and altered boundary conditions.

All other experimental parameters, including capsule excitation frequency, contact configuration, data acquisition protocol, and signal processing pipeline, were kept identical across the baseline and fluid‐loaded experiments in order to isolate the effect of intraluminal fluid conditions.

#### 4.2.2 Blinded Experimental Protocol

The blinded experiments were conducted using the ex vivo porcine‐colon platform. To minimize unintentional bias, all validation experiments were conducted using a randomized and blinded protocol with strict separation of roles between specimen preparation, data acquisition, and data analysis. For each colon specimen, multiple predefined measurement locations were marked prior to data acquisition.

Baseline vibration measurements were first collected at all marked locations before agar injection, providing normal tissue reference data across multiple anatomically distinct regions of the same specimen. A randomly selected subset of locations (approximately 30% of the marked points) was then injected with agar to generate artificial lesions, while the remaining locations served as internal controls. The selection of injection sites was not disclosed to the operator responsible for capsule placement and vibration data acquisition.

Vibration data were acquired sequentially at all marked locations under identical experimental conditions. At each location, measurements were repeated to assess repeatability and reduce measurement variability. Each colon segment was treated as an independent biological specimen, and experiments were performed across multiple specimens harvested from different anatomical regions to account for both intra‐ and inter‐specimen mechanical variability.

All data analysis was conducted only after completion of all experiments. Lesion labels were assigned after signal processing and feature extraction, ensuring that data analysis was performed without access to lesion presence or location information during data acquisition and initial analysis.

### 4.3 Statistical Analysis

As described above, classification performance was quantified using confusion‐matrix metrics, including accuracy, FPR, and FNR. Baseline normal tissue was treated as the inlier class, whereas mechanically abnormal regions were treated as outliers. The FPR thus denotes the proportion of abnormal samples incorrectly retained as normal (inliers), while the FNR denotes the proportion of normal samples rejected as abnormal (outliers). Where applicable, statistical significance was assessed at a threshold of p<0.05.

Prior to OCSVM classification, features were extracted from the magnitudes of the pre‐processed windows of triaxial acceleration signals, as described in Section 2. The resulting features were z‐score standardized using baseline statistics, and PCA was then applied to reduce the feature set to three principal components, which were used as inputs to the OCSVM and also supported visualization in the reduced feature space. Each triaxial recording at the marked sites was acquired over a sufficiently long duration, given the slow nature of peristaltic driving, and was segmented into two‐second windows. In the in vitro platform, 441 windowed signals were obtained from the normal phantom, while the abnormal dataset comprised 657 signals (219 for each lesion type: soft, medium, and hard). In the ex vivo porcine‐colon experiments, 1,250 signals were acquired from multiple normal‐tissue locations, while the blinded dataset (contained 4,987 signals) comprised recordings from soft, medium, and hard lesion sites under varying fluid‐load conditions, together with an additional 250 signals collected from multiple normal‐tissue sites. All performance metrics were computed at the window level, with aggregated statistics reported across experimental conditions.

## Author Contributions

Y.L. conceived the idea and designed the research. X.F., A.B., and B.T. developed the prototype, performed the experiments, and collected the data. K.A. analyzed the data and developed the AI system. Y.Y. formulated the mathematical model and conducted parameter identification. Y.L. and S.P. supervised the project. X.F., Y.Y., K.A., A.B., B.T., Y.L., and S.P. wrote the manuscript. All authors discussed the results and contributed to the final revision of the manuscript.

## Conflicts of Interest

The authors declare no conflicts of interest.

## Data Availability

All data needed to evaluate the conclusions in the paper are present in the paper.
